# Proof of concept study of mass cytometry in septic shock patients reveals novel immune alterations

**DOI:** 10.1038/s41598-018-35932-0

**Published:** 2018-11-23

**Authors:** Morgane Gossez, Thomas Rimmelé, Thibault Andrieu, Sophie Debord, Frédérique Bayle, Christophe Malcus, Françoise Poitevin-Later, Guillaume Monneret, Fabienne Venet

**Affiliations:** 1 Hospices Civils de Lyon, Immunology Laboratory, Edouard Herriot Hospital, Lyon, France; 2 EA 7426 “Pathophysiology of Injury-Induced Immunosuppression – PI3”, Université Claude Bernard Lyon 1/bioMérieux/Hospices Civils de Lyon, Edouard Herriot Hospital, Lyon, France; 3 Hospices Civils de Lyon, Anesthesia and Critical Care Medicine Department, Edouard Herriot Hospital, Lyon, France; 4 Univ Lyon, SFR Biosciences, ENS de Lyon, Inserm US8, CNRS UMS344, UCBL, Lyon, France

## Abstract

Innovative single cell technologies such as mass cytometry (CyTOF) widen possibilities to deeply improve characterisation of immune alterations mechanisms in human diseases. So far, CyTOF has not been used in sepsis – a condition characterized by complex immune disorders. Here, we evaluated feasibility of CyTOF analysis in patients with septic shock. We designed a mass cytometry panel of 25 extracellular markers to study mononuclear cells from 5 septic shock patients and 5 healthy donors. We explored single-cell data with global and specific unsupervised approaches such as heatmaps, SPADE and viSNE. We first validated relevance of our CyTOF results by highlighting established immune hallmarks of sepsis, such as decreased monocyte HLA-DR expression and increased expressions of PD1 and PD-L1 on CD4 T cells and monocytes. We then showed that CyTOF analysis reveals novel aspects of sepsis-induced immune alterations, *e.g*. B cell shift towards plasma cell differentiation and uniform response of several monocyte markers defining an immune signature in septic patients. This proof of concept study demonstrates CyTOF suitability to analyse immune features of septic patients. Mass cytometry could thus represent a powerful tool to identify novel pathophysiological mechanisms and therapeutic targets for immunotherapy in septic shock patients.

## Introduction

Sepsis is defined as a life-threatening organ dysfunction caused by a dysregulated host response to infection^1^. The World Health Organization has recently acknowledged sepsis as a major health care issue^2^. Indeed, incidence of septic syndromes is continuously increasing, affecting more than 35% of patients hospitalized in intensive care units (ICU) in Europe^3^. Moreover, septic shock, *i.e*. subset of sepsis with acute circulatory failure, is associated with mortality up to 45% in the ICU despite improvements in early care of organ dysfunctions^4^.

Thus, research effort focuses on finding specific treatment for sepsis. In the last few years, there has been renewed interest for the role and underlying mechanisms of sepsis-induced immunosuppression. Indeed, it has been demonstrated that immune alterations observed after sepsis were associated with increased risk of secondary infections and mortality^5^. Recently, new therapeutic approaches targeting these alterations in patients with persistent immunosuppression have emerged. Relevance of this strategy has been lately illustrated by encouraging results from a phase II trial using interleukin 7 as an immunoadjuvant therapy targeting adaptive immunity defects in septic shock patients^6^. Nevertheless, pathophysiology of immune dysfunctions developed after sepsis remains incompletely understood. Yet, exhaustive characterisation of these immune alterations is fundamental to highlight potential new targets and biomarkers, which would allow development of immune-based therapy of this hitherto deadly disease.

Such comprehensive studies of sepsis-induced host response and immune alterations were long limited by technology issues. Emergence of innovative single cell technologies widens possibilities to improve our understanding of sepsis pathophysiology in humans^7,8^. Particularly, mass cytometry (CyTOF) is a promising alternative to multicolour flow cytometry allowing to measure above 40 parameters simultaneously^9^. Indeed, CyTOF uses monoclonal antibodies conjugated with metal isotopes, avoiding common autofluorescence and spectral overlap issues occurring with conventional flow cytometry. These properties allow broad phenotypic exploration of several immune populations at once.

In this study, we used for the first time CyTOF technology to analyse immune alterations in a cohort of patients with septic shock. As a preliminary proof of concept study, the main objective was to evaluate feasibility and opportunities provided by mass cytometry in this clinical context. We designed a mass cytometry panel comprising 25 antibodies to explore mononuclear cell features from septic shock patients and healthy donors. Unsupervised analysis strategies such as SPADE^10^ and viSNE^11^ were used to highlight novel aspects of immune alterations developed after septic shock.

## Results

### Set up of blood processing and mass cytometry analysis

First, we tested 4 freezing protocols which differed in freezing and thawing media and in freezing/thawing steps (see Supplementary Table S1). We observed similar yield in number of cells available after thawing between the 4 protocols. However, protocol #4 was more convenient and easy to use, and included the use of DNAse during the thawing step, which allowed to avoid cell aggregates, especially in septic shock patients’ samples. Protocol #4 was thus selected to build the biobank.

**Figure 1:**
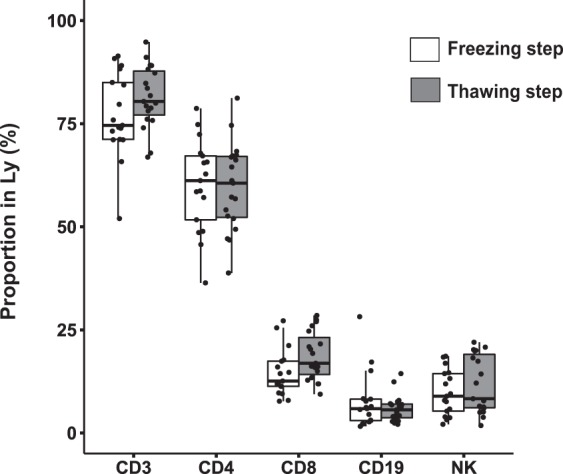
No deletion of a particular lymphocyte sub-populations is noted during the whole mass cytometry analysis process. Before freezing (white boxes) and after thawing (grey boxes) processes, proportions of lymphocyte sub-populations in mononuclear cells were analysed by flow cytometry in 10 samples from healthy volunteers and 11 samples from septic shock patients.

Next, we collected 10 samples from healthy volunteers and 11 samples from septic shock patients included at day 3 after the onset of septic shock. As significant loss in number of cells is commonly observed during freezing/thawing process^7^, we first assessed the recovery of proportion of lymphocyte subpopulations measured by flow cytometry before freezing and after thawing steps. Using the analytical conditions described above to process our samples, no loss of any particular lymphocyte population was noted, allowing further data analysis (Fig. 1). The number of viable cells available after the whole process was adequate for a relevant mass cytometry analysis.

### CyTOF analysis in septic shock patients

Due to limited access to CyTOF platform, we restrained the number of samples analysed by mass cytometry to 5 healthy volunteers and 5 septic patients. As our mass cytometry panel was designed for studying mononuclear cells, we selected the 5 septic shock samples presenting with the highest proportion and number of mononuclear cells in peripheral blood mononuclear cells (PBMC) layer after Ficoll. Clinical and biological data for the 5 septic shock patients are presented in Supplementary Table S2. Median [IQR] age of the healthy volunteers (HV) was 61 [53–63] years old and 40% were male.

**Figure 2:**
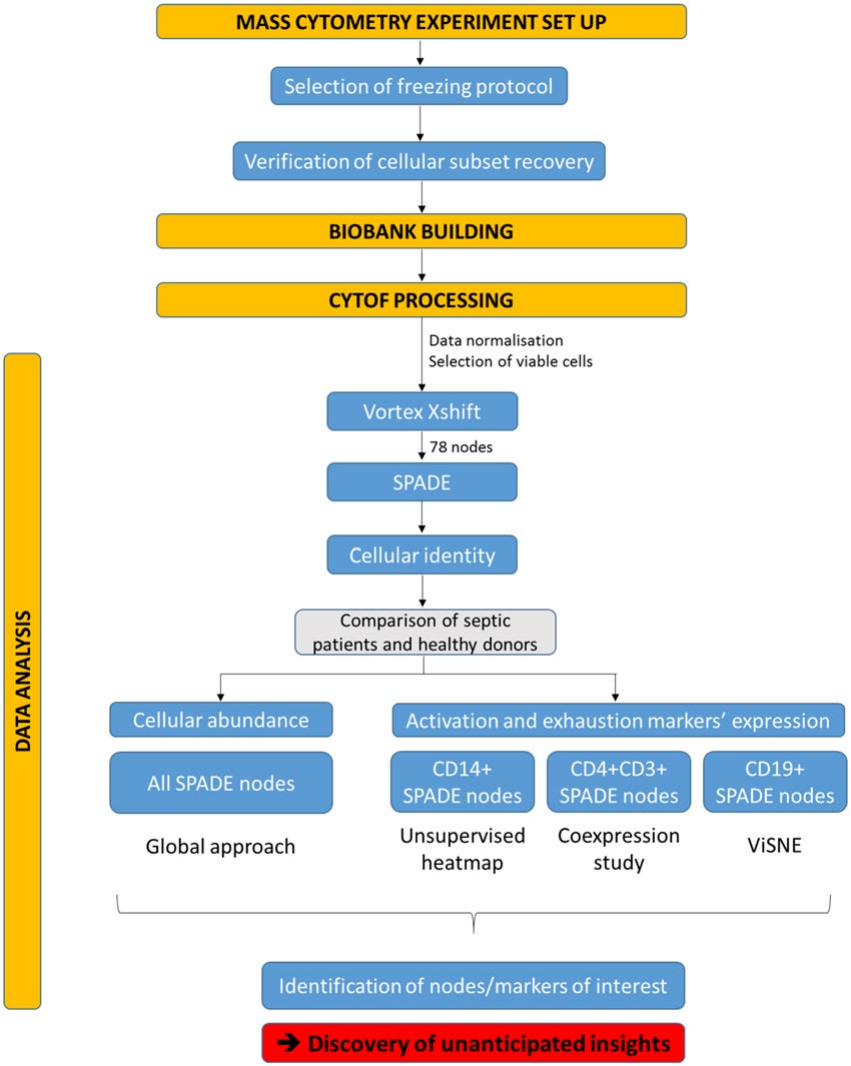
Analysis pipeline used throughout the study. The study was built through several steps: set up of mass cytometry analysis, process of samples on mass cytometer and data analysis. In this last step, several multidimensional data analysis tools were used, such as SPADE and viSNE, to highlight differences between septic shock patients and healthy donors in terms of cellular abundance and expression levels of exhaustion, activation and differentiation markers.

**Figure 3:**
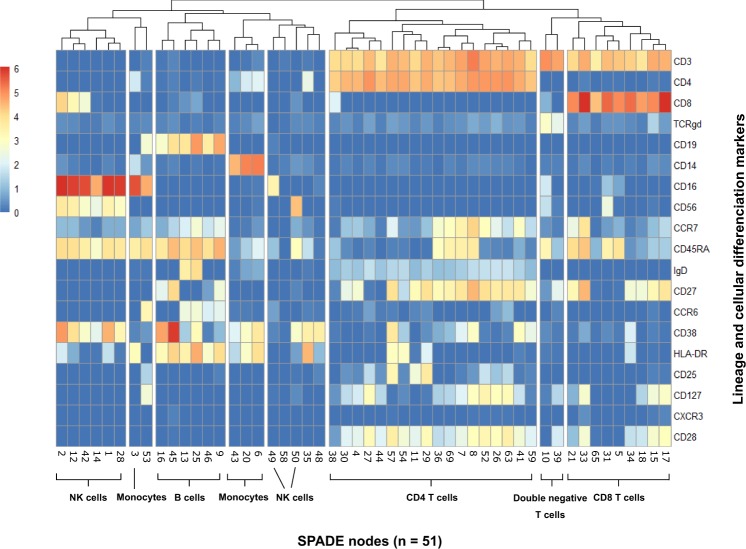
Expression of phenotypic markers used for node characterisation. Median expression for each marker was averaged from all 10 samples for each of the 51 nodes. Results are shown on heatmap after hierarchical clustering. Colour intensity specifies the expression level. Combined expression of several markers allowed to identify characteristic leukocyte populations, but also rare cell subsets. For instance, two populations of CD3^+^ cells but double negative CD4^−^CD8^−^ were noted, both expressing TCRγδ, but showing different features, such as the expression level of CD8, CD16 and CD56.

The analysis pipeline used throughout the study is illustrated in Fig. 2. To characterize and visualize mononuclear cells’ subpopulations in the observational cohort, we ran a SPADE clustering analysis. SPADE algorithm first downsamples data to capture rare populations. Then it hierarchically clusters phenotypically similar cells into “nodes” and represents these nodes and their relationships in a minimum spanning tree format (*i.e*. SPADE tree). The main issue with SPADE analysis is the definition of the optimal number of cell clusters. This is difficult to determine empirically. Firstly, to address this problem, we determined with X-shift algorithm the optimized cluster number for our dataset, as described elsewhere^12^. X-shift algorithm uses weighted *k-*nearest-neighbour density estimation to define the optimal number of cell clusters in high dimensional data. For this, X-shift evaluates the impact of different numbers of nearest neighbours on the number of cell clusters, with the optimal number of cell clusters defined at the “switch point” between under-clustering and over-fragmenting data. In our dataset, optimal cluster number was found at 78. Secondly, from this result we ran a SPADE analysis seeking 78 nodes. For further analysis, only nodes with an average proportion ≥0.5% of total cells were selected, in order to exclude sparse clusters where low cell numbers could potentially increase the error of reported medians. This threshold resulted in exclusion of approximately 6% of total cells, thus retaining 51 nodes for further analysis (see Supplementary Fig. S1A). The individual SPADE trees for control and septic samples are shown in Supplementary Fig. S1B. We thus finally identified cell types for each of the 51 cell nodes drawing a heatmap depicting the mean expression of 19 lineage or cellular differentiation markers (Fig. 3).

### Node distribution shows different proportions in septic shock patients compared to healthy donors

**Figure 4:**
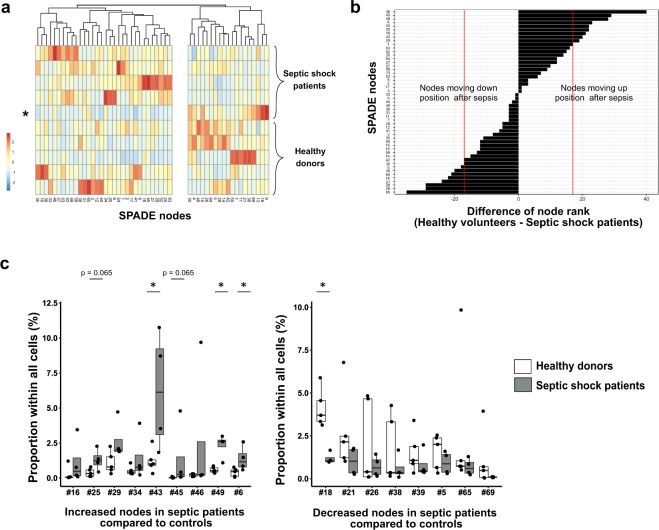
Significant variations in node proportions are observed after septic shock. (**A**) For the 5 healthy donors and 5 septic shock patients, individual proportions of each SPADE node were plotted onto a heatmap. The red and blue colours indicate increased and decreased proportions respectively. Order of nodes was determined by non-supervised hierarchical clustering. On the right side of the heatmap were clustered nodes that seemed decreased in septic patients compared to controls. On the left side were clustered nodes that looked increased in septic shock patients. Septic patient presenting mHLA-DR > 20,000 AB/C and excluded from further analyses is indicated by a star. (**B**) To highlight SPADE nodes with important variations in cell proportions between healthy donors and septic patients, nodes were ranked by descending order according to their average proportions in controls (n = 5) and in septic shock patients (n = 4). The rank difference between healthy volunteers and septic patients was calculated and plotted for each node. A difference (controls - septic patients ranks) under 0 reflected a decrease of cell number within the node in septic patients, whereas a positive difference suggested an increased node proportion in septic patients compared to healthy donors. Arbitrary, proportion variation was considered non-negligible when a node’s rank differed more than 1/3 of total node number, reflecting a gain or loss of more than 17 positions (red lines). (**C**) Potentially increased (left plot) or decreased (right plot) nodes’ frequencies were plotted for septic shock patients (n = 4; grey boxes) and healthy donors (n = 5; white boxes) samples as boxplots with individual values. Node proportions between controls and septic shock patients were compared using Mann-Whitney tests. *p < 0.05.

Next, we compared node proportions between septic patients and healthy volunteers. We drew a new heatmap representing proportions of each node in each individual (Fig. 4A). Clustering highlighted two groups of nodes, including respectively nodes with increased and decreased proportions in septic patients compared to healthy donors. Intriguingly, one septic shock patient showed a pattern of node’s proportions very similar to healthy volunteers’. When analysing clinical data for this patient, result of HLA-DR expression on monocyte (mHLA-DR) was 20,445 antibodies/cell (AB/C) whereas the four other septic patients had a mHLA-DR < 10,000 AB/C. As we focused on sepsis-induced immunosuppression, we decided to exclude this patient in further analyses, to avoid a risk of masking potential differences between controls and septic patients in this small cohort.

To identify nodes with the most important variations in cell proportion between HV and septic shock patients, we ranked nodes according to their average proportions in HV and in patients respectively. For each node, the rank difference between HV and septic patients reflected increased or decreased proportion after septic shock. For further analysis, we selected only the nodes with a rank difference of more than 1/3 of total node number (Fig. 4B). Thus, we identified 9 increased nodes and 8 decreased nodes after septic shock. Interestingly, all 8 decreased nodes were CD4 or CD8 T cells while the 9 increased nodes were mainly non-T cells. Especially, 4 nodes were B cell clusters, highlighting a potential enrichment after septic shock in this usually sparsely represented leukocyte subpopulation.

So as to confirm these changes between groups, we analysed node frequencies across individuals in the 17 selected nodes, especially to get rid of average variations that could hide high inter-individual variations. Figure 4C shows significant proportion increases in nodes #6, 43 and 49 in septic shock patients (p < 0.05; Mann-Whitney). These 3 nodes appeared to be conventional monocytes CD14^+^HLA-DR^+^, particular monocytes CD14^+^HLA-DR^low^ and a population of CD56^−^CD16^+^ cells respectively. A significant decrease in node #18 representing specific effector memory CD8 T cells was also observed in patients (p < 0.05; Mann-Whitney). Finally, we noted a trend towards increased proportions of nodes #25 and 45 (p = 0.065; Mann-Whitney), which represented naïve B cells and plasmablasts respectively.

### Specific phenotype changes are observed on leukocytes after septic shock

In order to highlight the broad potential of mass cytometry data analysis, we next investigated phenotypic changes in activation and exhaustion markers’ expression, using three different approaches: unsupervised heatmap, coexpression study and viSNE algorithm. These approaches were applied on monocytes, CD4 T cells and B cells respectively, selecting SPADE nodes according to the expression of lineage markers CD14, CD3/CD4, and CD19 (Fig. 2).

#### Uniform modifications in monocyte expression of 4 specific markers after septic shock

**Figure 5:**
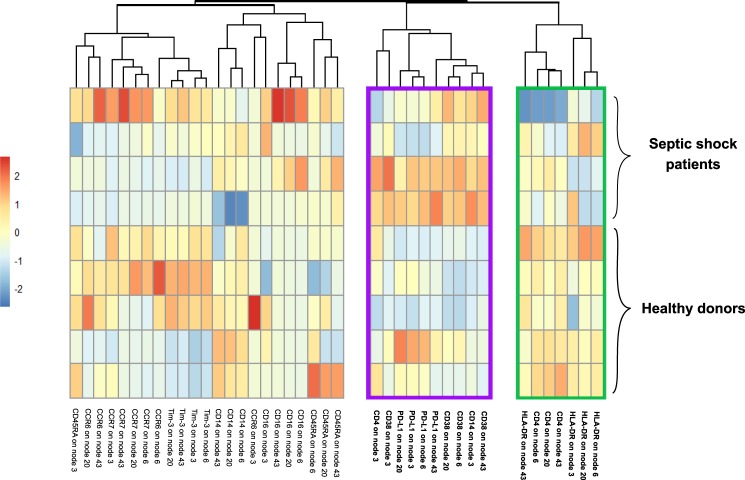
Expression of 4 monocyte markers is regulated after septic shock. After normalisation, median expression of phenotypic markers on each SPADE node of monocytes was shown on heatmap after hierarchical clustering for 4 septic shock and 5 healthy donors. The red and blue colours indicate over- and under-expressed markers respectively and the colour intensity specifies the expression level. Rectangles in green and purple indicate respectively markers down-regulated and up-regulated on particular nodes in septic shock patients compared to controls.

First, we were interested in monocyte activation and immunoregulatory profile after septic shock. We drew a heatmap of individual expression of activation markers (CD4, CD14, CD16, CD38, CCR6, CCR7, CD45RA) and immune checkpoint inhibitors (PD-L1 and Tim-3) on each of the 4 monocytes nodes from SPADE (*i.e*. nodes #3, #6, #20 and #43) (Fig. 5). PD1 and Lag-3 were not expressed at all on monocytes. Unsupervised clustering revealed three groups of markers depending on their modulation of expression on monocytes after septic shock. Interestingly, markers with increased (purple rectangle) or decreased (green rectangle) expression in septic patients were uniformly modulated on all monocyte nodes. Indeed, septic patients presented with an increased expression of CD38 and PD-L1, as well as a decreased expression of HLA-DR and CD4 on all septic monocyte nodes compared to healthy donors, except on node #3 for the latter parameter.

#### High heterogeneity in PD1+ CD4 T cells after septic shock, without coexpression of other immune checkpoint inhibitors

**Figure 6:**
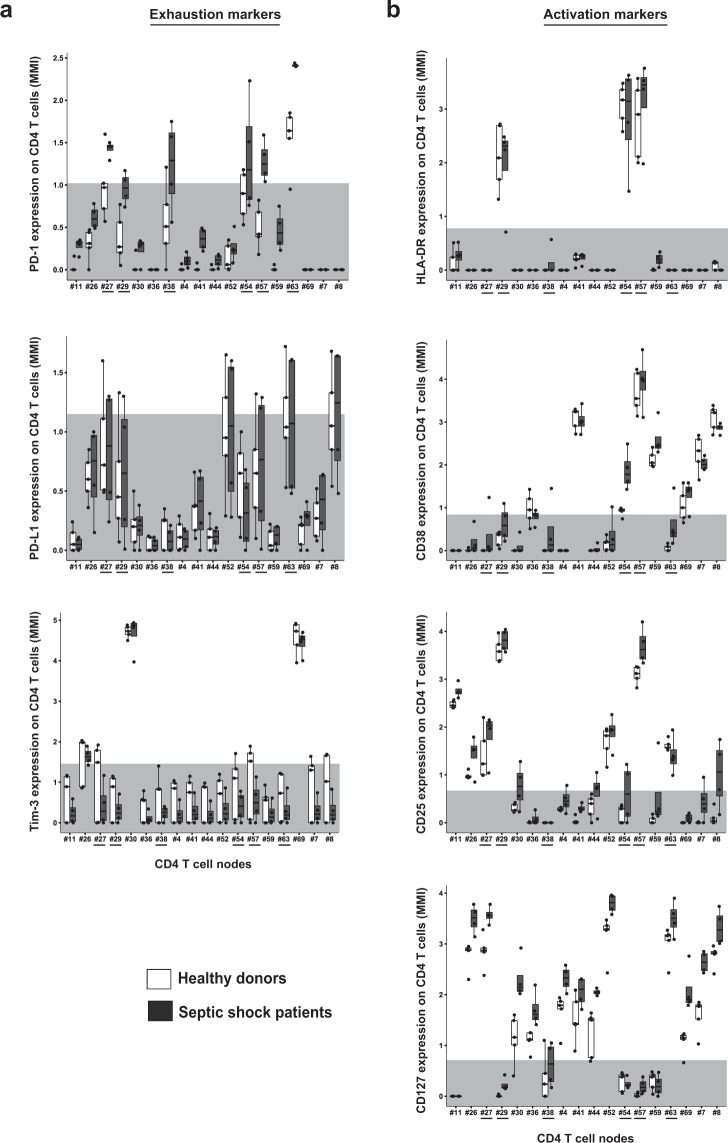
PD1 expressing CD4 T cells show high heterogeneity after septic shock and do not co-express other immune checkpoint inhibitors. Expressions of several exhaustion (**A**) and activation (**B**) markers on CD4 T cell nodes from SPADE were compared in septic shock patients (n = 4, dark grey boxes) and healthy volunteers (n = 5, white boxes). The light grey areas represent global median expression on CD4 T cells from mass-minus-one control, characterising the threshold of positive expression for each marker. PD-1 expressing nodes are highlighted underlined.

Considering the expanding importance of immunostimulating therapies targeting immune checkpoint inhibitors, we studied expression of PD1, PD-L1 and Tim-3 on the 18 CD4 T cell nodes from SPADE (Fig. 6A). This was performed by representing individual values and box-plots of MFI of each marker on all CD4 T cell nodes in HV and patients. In addition, showing background signal measured on a mass-minus-one (MMO) sample for each marker was helpful to compare co-expressions. No difference of Tim-3 or PD-L1 expressions was observed between HV and septic patients. In HV, PD1 was systematically expressed on a unique node (#63). However, in septic shock patients PD1 exhibited increased expression on node #63 and appeared on several other nodes, *i.e*. nodes #27, #29, #38, #54 and #57. Co-expression of PD1 and PD-L1 was rarely observed. Interestingly, Tim-3 was expressed only on 2 nodes, and was not co-expressed with neither PD1 nor PD-L1. Next, we studied expression of HLA-DR, CD38, CD25 and CD127 on these five PD1^+^ nodes to investigate whether the increased expression of PD1 was associated with expression of activation markers (Fig. 6B). There was almost no expression of these markers on node #38, which was the only node of double positive CD4^+^ CD8^+^ T cells. Nodes #27 and #63 did not express HLA-DR nor CD38, but were CD25^+^ and showed high expression of CD127, suggesting potential resting memory cells. Node #54 had a profile of activated effector cells, HLA-DR^+^ CD38^+^ CD25^low^ and CD127^−^. Finally, high expressions of HLA-DR, CD38 and CD25 on nodes #29 and #57 were observed, in association with a loss of CD127 expression. This latter phenotype could be a feature of either activated effector CD4 T cells or regulatory T cells. None of these nodes had significant increased proportion in septic shock patients compared to healthy volunteers.

#### B cell commitment to plasma cell differentiation in septic shock patients

**Figure 7:**
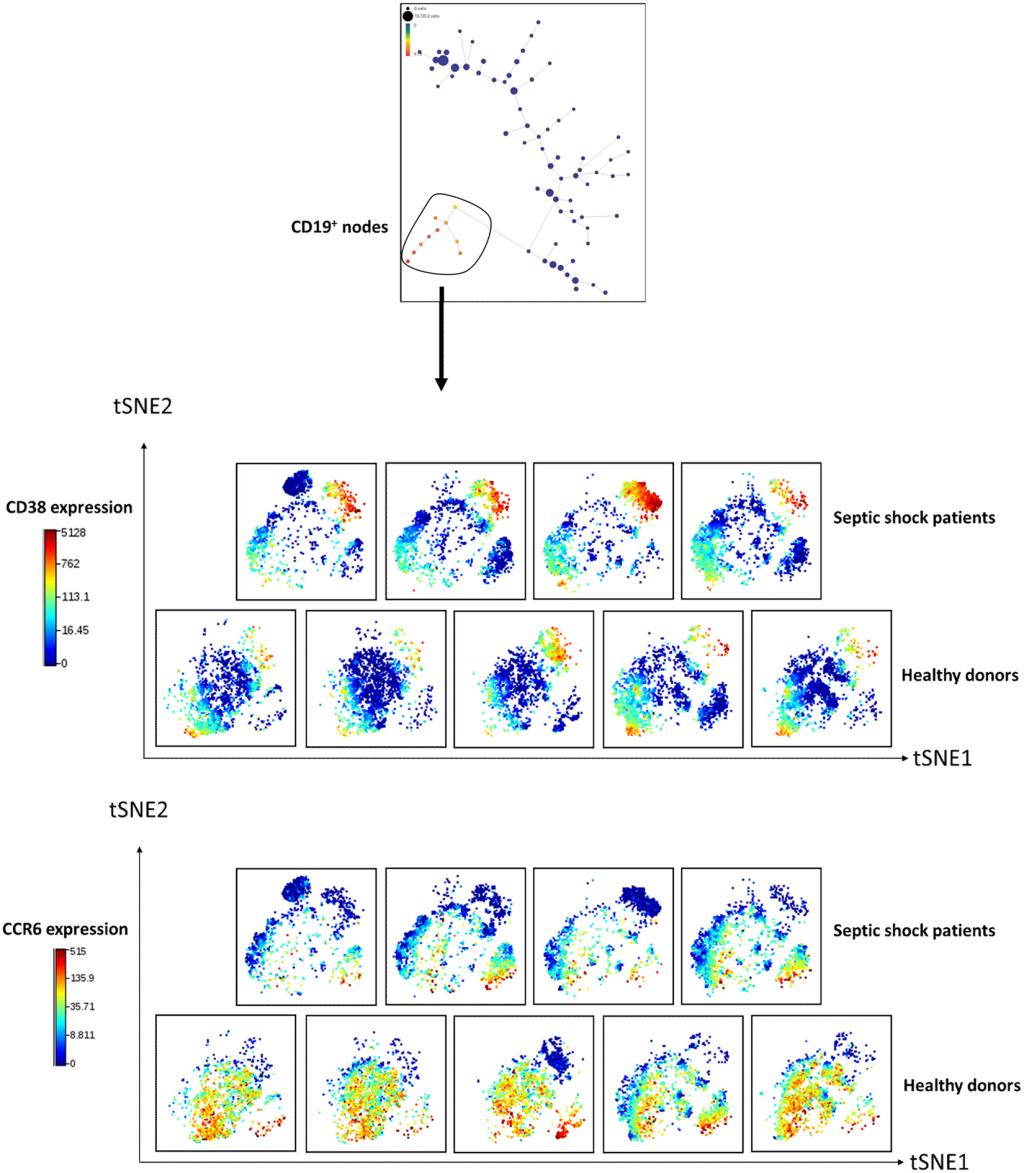
B cells shift towards plasma differentiation after septic shock. Cells from the 10 CD19^+^ nodes from SPADE tree (red nodes) were selected before clustering with the viSNE algorithm. Individual viSNE plots are shown for septic patients (n = 4) and healthy donors (n = 5). ViSNE is a visualization tool based on the t-Distributed Stochastic Neighbor Embedding (t-SNE) algorithm. ViSNE establishes the two dimensional representation of single-cell data sets that best preserves their respective local and global geometry. ViSNE plots represent individual cells in a visual similar to a scatter plot, while using all pairwise distances in high dimension to determine each cell’s location in the plot. tSNE algorithm provided each cell with a unique x- and y-coordinate (tSNE1 and tSNE2) according to each cell’s expression of 19 parameters of the CyTOF panel. The relative positions of cells on the two-dimensional plot thus reflect their similarity in terms of expression pattern for the 19 parameters included in the panel. In the current analysis, we used color as a third dimension to visualize specific markers expression levels on each cluster. B cell expressions of CD38 and CCR6 are shown on the figure. Red color intensity represents high expression level of the indicated marker. Blue color intensity represents low expression level of the indicated marker. Plots were visually compared between septic patients and controls to highlight differences in marker intensity and cellular distribution.

Finally, given the enrichment in B cell populations in septic shock patients, we went further in B cell study. We ran a viSNE analysis on all CD19^+^ cells from SPADE tree (Fig. 7). ViSNE is a well-established CyTOF analysis tool that displays high dimensional data on a two-dimensional map. Cells are plotted on a continuum of expression with phenotypically related cells clustered together, often manifesting as “islands”, which can be identified by colouring cells according to their expression of specific marker. A gap in island distribution between two conditions can indicate an altered cell type or phenotype. Here, viSNE maps from patients and controls did not show the same pattern, suggesting a reorganisation of B cell subpopulations after septic shock. To further investigate this observation, cells were coloured on viSNE plots according to cell surface expression of CD38 or CCR6, two markers of B cell differentiation^13^. We observed that, in both healthy donors and septic shock patients, CD38^+^ and CCR6^+^ cells did not present same location on the viSNE map, indicating that they belonged to different cell subtypes. Moreover, we could highlight an increase of CD38^+^ cells in septic patients, as well as a decrease of CCR6^+^ cells compared to healthy donors. These observations suggested a shift from B cells to plasma cell differentiation in septic shock patients.

## Discussion

Exhaustive characterisation of sepsis-immune alterations has not been completed yet, limiting the identification of potential new specific targets of immunotherapies. In this study, we hypothesized that high dimensionality afforded by CyTOF could be a useful tool to detect novel and unexpected immune features in septic shock patients. To date, very few studies have used CyTOF technology to explore infectious diseases in clinical cohorts^14–16^ and none specifically in septic shock. So far, CyTOF has been more often employed as an exploration tool of *in vitro* models of infections^17–19^. Here, we set up a proof of concept study with a mass cytometry panel of 25 markers, in order to compare immune phenotype of mononuclear cells from septic shock patients and healthy volunteers.

CyTOF represents a powerful high dimensional single-cell analysis technique that overcomes major limitations of flow cytometry. In particular, through the use of antibodies coupled to rare elemental metal isotopes, mass cytometry can detect discrete isotope peaks without significant overlap. This significantly ameliorates the need for compensation, a major limiting factor of flow cytometry. In addition, mass cytometry has no “autofluorescence” equivalent. This limits the loss of resolution that could be observed in flow cytometry because of high background level, thus making data analysis easier. Finally, signal intensity is more homogeneous with isotopes than fluorochromes, which sometimes show important brightness differences between the highest and dimmest, complicating panel design. This leads to higher multiplexing capability and easier panel design in CyTOF than in flow cytometry, enabling simultaneous measurement of several tens of markers. Thus, CyTOF appears more appropriate than conventional flow cytometry to take over high dimensional data analysis.

However, cell loss during freezing steps and CyTOF acquisition, high amount of cells needed, low throughput and complex high dimensional data analysis are many hurdles that might prevent the use of CyTOF in clinical studies of patients with septic shock^7^. Here, we show that pre-analytical process did not lead to any specific cell subset depletion among mononuclear cells and that final cell number was adequate for a relevant mass cytometry analysis. Moreover, reliability of our data was validated by non-supervised approaches used throughout the pipeline analysis. First, expression of lineage and differentiation markers clustered SPADE nodes in coherent distinct leukocyte subsets leading to identification of cell types corresponding to each node. Then, non-supervised approaches were able to highlight some established hallmarks of septic shock immune alterations. Particularly, in septic shock patients we observed a decreased expression of HLA-DR on monocytes^20^, an increase of CD14^+^HLA-DR^low^ monocyte proportions^21^ and an overexpression of PD1 and PD-L1 on CD4 T cells and monocytes respectively^22^.

As hypothesised, CyTOF single cell data analysis allowed discrimination of many cell subsets, enabling to study specific rare populations and to highlight new features of sepsis-induced immunosuppression. First, a global approach on all SPADE nodes led to the identification of particular leukocyte subpopulations with modifications of their proportion after septic shock. These changes would have probably been missed with bulk analysis considering low abundance of the cell populations described. For example, we noted an increased proportion of CD3^−^CD19^−^CD14^−^CD16^+^CD56^−^ cells in septic shock patients. This population could represent an interesting subtype of CD56 negative NK cells, described as an aberrant functionally altered NK subset, as they present with low capacity of proliferation, cytokine secretion, degranulation and expression of activation receptors. They were observed with elevated levels in individuals chronically infected with human immunodeficiency virus-1 (HIV-1) and hepatitis C virus (HCV)^23^ but, to our knowledge, never described in septic shock. While this NK population could be of interest in sepsis-induced immunosuppression physiopathology, we cannot exclude the possibility that these cells represent some immature granulocytes frequently observed in septic shock patients’ samples. As our mass cytometry panel did not contain markers dedicated to granulocyte characterization, we were not able to discriminate the two hypotheses, which now deserve to be further studied in septic shock patients. We also noted a trend toward B cell enrichment among mononuclear cells in septic shock patients, which had been very recently described by standard flow cytometry^24^.

Then, we studied changes in cellular phenotypes after septic shock through more specific approaches. First, we focused on monocyte SPADE nodes. Remarkably, among the 9 markers used in the heatmap analysis, unsupervised clustering highlighted an immune signature of 4 markers (HLA-DR, CD4, CD38 and PD-L1) differentially expressed in healthy volunteers and septic shock patients. This uniform response might be used as potential biomarker of sepsis outcomes and then should be confronted to clinical recovery in larger cohorts, e.g. development of hospital acquired infection or mortality. Next, we were interested in the characterisation of CD4 T cell nodes expressing PD1, as many novel immunostimulating therapies target this molecule. Increase of PD1 expression did not occur on all CD4 T cell subsets after septic shock. Interestingly, CD4 T cells exhibiting PD1 upregulation did not coexpress other immune checkpoint inhibitors Tim-3 and PD-L1. Furthermore, increased expression of PD1 was observed on CD4 T cells presenting with different activation status, as some PD1^+^ cells clearly exhibited activated phenotypes whereas some corresponded to resting memory T cells. Here, CyTOF revealed to be a powerful tool to highlight and study cellular heterogeneity, as demonstrated recently for exhausted CD8 T cells in other clinical contexts^16^. In particular, our results imply that exhaustion phenomenon might not occur in all cell subsets after septic shock, or at least might not be systematically associated with PD1 overexpression. These observations should be kept in mind and further explored when considering anti-PD1 strategy to treat septic patients. They strengthen the need of developing biomarkers of patients’ immune status before using immunotherapies. Finally, when focusing on CD19^+^ cells, we observed a shift of B cell subsets towards plasma cell differentiation after septic shock. Thus we confirmed a feature of sepsis described recently for the first time by our group^24^. It was striking to be able to detect this phenomenon in such a small cohort.

The aim of the present study was to assess feasibility and opportunities provided by CyTOF use in sepsis clinical cohorts. Thus, by nature, as a proof of concept study, our work has some limitations. In particular, we included small numbers of HV and septic shock patients. However, even in such a small cohort, non-supervised analyses of high dimensional data strikingly succeeded in highlighting both well-established (*e.g*., low mHLA-DR) and novel characteristics (*e.g*., increased plasma cells) of sepsis-induced immunosuppression. This illustrates the power of CyTOF technology. Furthermore, CyTOF was able to identify one septic patient with a singular immune pattern compared to others. This was confirmed by unusually high mHLA-DR expression measured by traditional flow cytometry. Again, this exemplifies CyTOF capacity to detect heterogeneity within septic shock patients’ immune patterns. Thus, upon analysis of larger cohorts, CyTOF could be relevant to reveal new features of sepsis pathophysiology on the one hand, and to stratify patients according to their immune status and trajectories over time on the other hand. This latter point is an absolute requirement in future studies evaluating immunomodulating therapies in clinical cohorts. Analysing data from more patients would also open the possibility to use new interesting algorithms and software tools, as Citrus, which are able to automatically identify immune signatures in cellular populations^12,25^.

Beyond the present study, there are several limiting factors for mass cytometry implementation in clinical studies. Indeed, CyTOF is a time consuming method, requiring 3 days of experiment from freezing to data acquisition. High dimensional data leads to days of data analysis, involving innovative bioinformatics tools such as SPADE and viSNE and representing analytical challenge for non-experts. However, mass cytometry represents a powerful tool to characterize immune alterations and observe the individual variability that must be targeted when developing immunomodulating therapies in context of personalised medicine. This has been illustrated in trauma context, where mass cytometry was used to link single-cell profiling of immunologic states to post-traumatic outcomes^26^, or in cancer context, revealing specific markers associated with melanoma immunotherapy response^27^. CyTOF could be relevant to highlight such immune signatures in sepsis.

In conclusion, in this study, we paved the way to the use of mass cytometry in sepsis clinical studies by demonstrating its reliability and feasibility in this context. We must now considered the use of standardized protocols on whole blood samples^28–30^ that would allow the use of this technology in multicentre studies. In addition, these preliminary results now deserve to be completed by larger studies combining extra and intracellular markers to explore signalling pathways and transcription factors, in order to progress in exhaustive characterisation of sepsis-induced immunosuppression.

## Patients and Methods

### Study population

This clinical study was conducted on septic shock patients admitted to the ICU of Edouard Herriot Hospital (Hospices Civils de Lyon, Lyon, France). This project is part of a global study on ICU-induced immune dysfunctions. It has been approved by our Institutional Review Board for ethics (“Comité de Protection des Personnes Sud-Est II”), which waived the need for written informed consent since this study was observational with low risk for the patients and no specific blood sampling procedure beside routine blood sampling was required (#IRB 11236). This study is also registered at the French Ministry of Research and Teaching (#DC-2008-509) and recorded at the Commission Nationale de l’Informatique et des Libertés. Oral information and non-opposition to inclusion in the study were mandatory and recorded in patients’ clinical files. This study is registered at NCT02803346.

Septic shock patients were identified according to the diagnostic criteria of the 1992 consensus definitions of the American College of Chest Physicians/Society of Critical Care Medicine^31^. Patients were excluded if under 18 years of age or presented with aplasia or pre-existent immunosuppression. Septic shock diagnosis was based on the combination of an identifiable site of infection with persisting hypotension — despite fluid resuscitation — and evidence of a systemic inflammatory response manifested by at least two of the following criteria: a) temperature >38 °C or <36 °C; (b) heart rate >90 beats/min; (c) respiratory rate >20 breaths/min; (d) white blood cell count >12,000/mm3 or <4,000/mm3. The onset of septic shock was defined by the beginning of vasopressor therapy.

Peripheral blood was collected in patients 3 days after the onset of septic shock during routine blood sampling procedure, since previous studies showed that this time-point corresponds to the nadir of sepsis-induced immunosuppression^5^. Clinical and biological parameters were collected, including demographic characteristics, date and cause of ICU admission, status at day 28 after inclusion, type of infection, comorbidities allowing to calculate Charlson comorbidity index^32^. Severity scores were recorded: the Simplified Acute Physiology Score II (SAPSII; range 0–194)^33^ and the Sepsis-related Organ Failure Assessment (SOFA; range 0–24)^34^.

Concomitantly, healthy volunteers were obtained from the blood bank Etablissement Français du Sang (EFS) in Lyon, France. According to EFS standardized procedures for blood donation, informed consent was obtained from healthy donors and personal data were anonymized at the time of blood donation and before the transfer of blood to the research lab.

### Sample preparation

PBMC from septic patients and healthy volunteers were isolated from 7 mL of blood freshly drawn on anticoagulated tubes with Lympholyte®-H Cell Separation Media (Cedarlane, Burlington, Canada) centrifugation. PBMC were immediately cryoconserved after isolation in Roswell Park Memorial Institute medium 1640 (RPMI) complemented with 4-(2-hydroxyethyl)-1-piperazineethanesulfonic acid (HEPES) and glutamine (Eurobio Abcys, Courtaboeuf, France) containing 40% AB human serum (SAB) and 10% dimethylsulfoxyde (DMSO) (Sigma-Aldrich, Saint-Louis, MO).

### Mass cytometry

Depending on the sample, between 0.5 and 2.5 million thawed PBMC were incubated during 5 min with a viability reagent (5 μmol/L Cell-ID™ Cisplatin; Fluidigm, South San Francisco, CA) and then washed twice in Dulbecco’s Phosphate-Buffered Saline (DPBS) containing 0.1% Bovine Serum Albumin (BSA). For staining, cells were incubated for 40 min at 4 °C in 100 μL DPBS/0.1% BSA containing antibody cocktail prepared with 25 antibodies listed in Supplementary Table S3. Pre-metal tagged antibodies were purchased from Fluidigm. Cells were washed twice in DPBS/0.1% BSA and then fixed overnight at 4 °C with 1 mL of MaxPar® Fix&Perm Buffer (Fluidigm) containing 1 µL of Cell-ID™ Intercalator-Ir 125 µM (Fluidigm). The following day, cells were washed twice with DPBS and finally once with deionized MaxPar® Water (Fluidigm). Cells were resuspended in 1×EQ Four Element Calibration Beads (Fluidigm Sciences) according to the manufacturer’s instructions. Samples were collected on a Helios mass cytometer (Fluidigm). Events were normalized prior to analysis as described elsewhere^35^. Normalized data were imported into Cytobank (Santa Clara, CA) and manually gated: first, singulets were identified as ^191^Ir^+ 193^Ir^+^ events and then viable cells as ^195^Pt negative events. Eventually, normalization beads were excluded by gating on 140Ce negative events (see Supplementary Fig. S2A). To analyse proportions of main PBMCs’ populations, cells were first gated according to their expression of CD3. CD4 and CD8 T cells were identified within CD3^+^ cells, while CD19^+^, CD14^+^ and CD16^+^/CD56^+^ non-T cells were used to classify B cells, monocytes and NK cells respectively (see Supplementary Fig. S2B). When studying individual expression of phenotypic markers, we used a mass-minus-one control to set positive threshold. It was constituted of thawed PBMC stained only with lineage markers CD3, CD4, CD8, CD14, CD19, CD16 and CD56.

### Flow cytometry

Proportions of CD4 and CD8 T cells, as well as monocytes in PBMC were determined by flow cytometry using a four-color monoclonal antibody panel consisting of CD45-FITC/CD4-PE/CD8-ECD/CD3-PC5 (Beckman Coulter, Hialeah, FL). A second antibody panel was used to measure B cells and NK cells’ proportions, using CD19-PB/CD3-FITC/CD16-CD56-PE/CD45-APC-AF750 (Beckman Coulter). Samples were run on a Navios flow cytometer (Beckman coulter). Listmodes were subsequently analysed using Kaluza software (Version 1.2). Briefly, in both panels, lymphocytes were first gated out of total events on a biparametric CD45/SSC dot-plot, as CD45^hi^ SSC^lo^ events. Then, T cell subpopulations were defined as CD4^+^ or CD8^+^ CD3^+^ lymphocytes while B and NK lymphocytes were characterized as CD3 negative cells expressing respectively CD19 and CD56/CD16.

### Data processing and analysis pipeline

The whole analysis pipeline is illustrated in Fig. 2.

#### X-shift

The 06/29/2017 version of VorteX was downloaded and manually gated singulet viable events from the 5 healthy donors and 5 septic shock samples were uploaded into the VorteX clustering environment^36^. The imported settings described previously were used^12^: minimal Euclidean length of the profile: 1.0, import maximum: 10,000 events, and merge all files into one dataset. All 24 parameters were selected for clustering. After the data were imported, the following clustering settings were used: numerical transformation: arcsinh (x/f), f = 5.0, noise threshold: 1.0, feature rescaling: none, normalization: none, distance measure: angular distance, clustering algorithm: X-shift (gradient assignment), density estimate: N nearest neighbors (fast), number of neighbors for density estimate (K): from 150 to 5, with 30 steps, and number of neighbors for mode finding (N): determine automatically. Following clustering, the elbow point for cluster number was calculated as K = 35, which corresponded to 78X-shift–defined clusters within the dataset.

#### Spanning-tree Progression Analysis of Density-normalized Events

SPADE analysis was performed on manually gated singulet viable events from the 5 healthy volunteers and 5 septic shock samples on Cytobank. Raw median intensity values were transformed to a hyperbolic arcsine (arcsinh) scale with a cofactor of 5. The target number of nodes was adjusted to 78 as defined with the X-shift clustering tool. Clustering channels included all 25 parameters except for CD138, because of elemental contamination in 2 samples. Percentage downsampling was 10%. SPADE trees for each individual across all 24 markers were downloaded from Cytobank. Intensity and cellular abundance of each node from each individual were exported for further analysis.

#### Visual Stochastic Network Embedding

All CD19^+^ nodes from SPADE tree were selected and subjected to viSNE analysis. Non-B lineage markers as well as CD138 were excluded from viSNE clustering. Equal event sampling was selected to allow comparison between individuals, using the lowest common denominator across samples, which was 3055 events. Other viSNE settings were applied: number of iterations 2000, perplexity 30, theta 0.5. Cellular phenotype was studied visualizing characteristic markers’ distribution and expression on viSNE plots and through generation of overlays on Cytobank.

#### Determination of node frequency variations between patients and controls

To select nodes with important variations in cell proportions between healthy donors and septic patients, nodes were ranked in each group by descending order according to their average proportions. A rank difference (control - septic patient ranks) under 0 reflected a decrease of cell proportion in septic patients, whereas a positive difference suggested an increased proportion in septic patients compared to healthy donors. Nodes with proportion variations differing of more than one tertile of total node ranks were selected and individual frequencies were compared between septic shock patients and healthy donors with appropriate statistical test.

### Statistical analysis

Statistical analyses, heatmaps and graphics exploring cellular abundance and expression were performed on RStudio software. Non-parametric Mann-Whitney tests were used to compare data between septic shock patients and controls. Non-parametric Wilcoxon paired test was used when comparison within the same sample was performed. Values from continuous variables are presented as medians with Q1-Q3 interquartile ranges [IQR] between brackets. p value < 0.05 was considered as significant.

## Electronic supplementary material

Supplementary Information
