# Supplementary material for: Proof of concept study of mass cytometry in septic shock patients reveals novel immune alterations
Source: Sci Rep. 2018 Nov 23;8:17296. doi: 10.1038/s41598-018-35932-0 (PMC6251894; doi:10.1038/s41598-018-35932-0)
Supplement: Supplementary file 1 — Supplementary Information [file 41598_2018_35932_MOESM1_ESM.docx]

**Proof of concept study of mass cytometry in septic shock patients reveals novel immune alterations**

Morgane Gossez, PharmD*^1,2^, Thomas Rimmelé, MD PhD^2,3^, Thibault Andrieu^4^, Sophie Debord, MD^3^, Frédérique Bayle, MD^3^, Christophe Malcus, PharmD PhD^1^, Françoise Poitevin-Later, PharmD^1^, Guillaume Monneret, PharmD PhD^1,2^, Fabienne Venet, PharmD PhD^1,2^

**SUPPLEMENTARY INFORMATIONS**

**Supplementary Table S1. Freezing and thawing protocols tested**

| **Steps** | **Protocol 1** | **Protocol 2** | **Protocol 3** | **Protocol 4** |
| --- | --- | --- | --- | --- |
| **Freezing medium (FM)** | SAB + 20% DMSO | RPMI  + 20% SAB + 20% DMSO | RPMI + ATB  + 50% SAB + 10% DMSO | RPMI  + 40% SAB + 10% DMSO |
| **Freezing step** | Add FM drop by drop in 1min | | | Add all FM volume at once |
|  | - Cell concentration of 1.5 – 2 x 10^6^ cells/ml - Dispense 1 ml cell suspension into cryovial - Immediately place cryovials into a freezing container (Mr Frosty) in a -80 ˚C freezer - On the following day, transfer the cryovials into standard freezer boxes | | | |
| **Thawing medium (TM)** | RPMI | RPMI + 10% SAB 37°C | SAB | RPMI + ATB + DNAse 37°C |
| **Thawing step** | - 1 ml TM drop by drop - Qsp 10 mL TM - 5 min resting - 2 washs | - 1 mL TM drop by drop - 10 min resting - Qsp 10 mL TM - 2 washs | - 1 mL TM drop by drop - 4 mL RPMI - 5 min resting - 2 washs | - 1 mL TM all at once - Qsp 10 mL TM - 2 washs |

RPMI: Roswell Park Memorial Institute medium complemented with hepes and glutamine; SAB: AB human serum; DMSO: Dimethysulfoxyde; ATB: antibiotics including penicillin, streptomycin and amphotericin B. Freezing/thawing protocols presented in this paper are in-house protocols from several laboratories in Lyon (Etablissement Français du Sang – blood bank in Lyon; Immunology Laboratory of Edouard Herriot Hospital) and Oxford (Peter Medawar Building for Pathogen Research).

**Supplementary Table S2. Clinical and biological data for septic shock patients**

| **N = 5** | |
| --- | --- |
| **Male** | 4 (80) |
| **Age (year)** | 70 [67-73] |
| **SAPSII score** | 55 [49-61] |
| **SOFA score** | 9 [7-9] |
| **Charlson score** | 2 [0-3] |
| **Type of admission** |  |
| Medical | 4 (80) |
| Emergency surgery | 1 (20) |
| **Type of infection** |  |
| Community acquired | 2 (40) |
| Nosocomial | 3 (60) |
| **Site of infection** |  |
| Pulmonary and respiratory | 1 (20) |
| Gastro and intra-abdominal | 1 (20) |
| Urinary | 2 (40) |
| Surgical site | 1 (20) |
| **Documentation of infection** |  |
| Clinics + surgery | 1 (20) |
| Microbiogically documented | 4 (80) |
| *BGN* | *3 (60)* |
| *CGP* | *1 (20)* |
| **Immune parameters at day 3** |  |
| mHLA-DR (AB/C) | 9,292 [7,219-9,716] |
| CD4 T cells (cells/µL) | 634 [536-750] |
| Tregs (% among CD4 T cells) | 7.4 [5.3-9.0] |

Five septic shock patients were included in this study. Values are presented as numbers (percentages) for categorical variables and as medians and [Q1-Q3] interquartile ranges for continuous variables. SAPSII: Simplified Acute Physiological Score II. SOFA: Sepsis-related Organ Failure Assessment score. BGN: Bacilli Gram Negative. CGP: Cocci Gram Positive. mHLA-DR: cell surface expression of HLA-DR on circulating monocytes. AB/C: number of antibodies bound per cell. Tregs: regulatory T cells. Normal value for mHLA-DR is > 15,000 AB/C, indicative of immunocompetence (1). In-laboratory age-matched values for CD4 T cell absolute count are 336–1126 cells/µL and 4–10% of total CD4 T cells for Tregs.

*Serum lactate level was not measured at admission for one patient.

**Supplementary Table S3. Mass cytometry antibody panel**

| **Elemental Isotope** | **Antigen Target (Human)** | **Clone number** |
| --- | --- | --- |
| 141Pr | CD196 (CCR6) | G034E3 |
| 143Nd | CD45RA | DX29 |
| 145Nd | CD4 | RPA-T4 |
| 146Nd | IgD | IA6-2 |
| 148Nd | CD14 | RMO52 |
| 149Sm | CD56 (NCAM) | NCAM16.2 |
| 150Nd | CD138 | DL-101 |
| 152Sm | TCRγδ | 11F2 |
| 153Eu | TIM-3 | F38-2E2 |
| 154Sm | CD3 | UCHT1 |
| 155Gd | CD279 (PD-1) | EH12.2H7 |
| 156Gd | CD183 (CXCR3) | G025H7 |
| 158Gd | CD27 | L128 |
| 159Tb | CD197 (CCR7) | G043H7 |
| 160Gd | CD28 | CD28.2 |
| 162Dy | CD8a | RPA-T8 |
| 165Ho | CD19 | HIB19 |
| 167Er | CD223 (LAG-3) | 3DS223H |
| 168Er | CD154 (CD40L) | 24-31 |
| 169Tm | CD25 (IL-2R) | 2A3 |
| 172Yb | CD38 | HIT2 |
| 173Yb | HLA-DR | L243 |
| 175Lu | CD274 (PD-L1) | 29E.2A3 |
| 176Yb | CD127 (IL-7Ra) | A019D5 |
| 209Bi | CD16 | 3G8 |

Pre-metal tagged antibodies were purchased from Fluidigm. Antibodies were used at final concentration of 1:100.

**
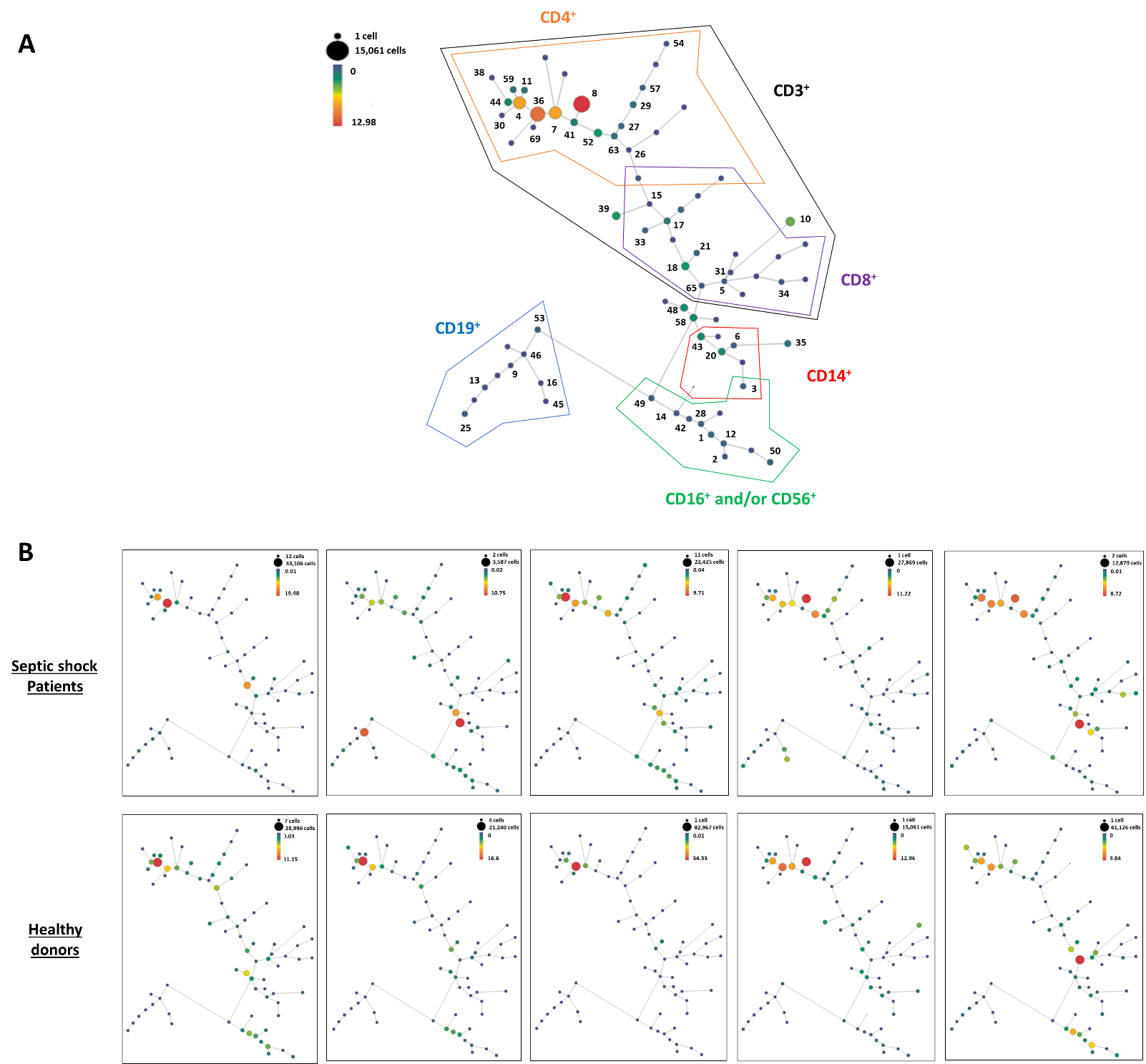
**

**Supplementary Figure S1. SPADE trees**

(**A)** Viable cells were parsed with SPADE into 78 nodes according to optimal clustering number determined with X-shift algorithm. 10% down-sampling was performed. All markers were used for clustering. Only nodes with an average proportion ≥ 0.5% of total cells (numbered nodes) were selected for further analysis in order to exclude sparse clusters in which low cell numbers could potentially increase the error of reported medians. Lineage marker expression was highlighted on each node, allowing identification of leukocyte subpopulations. (**B**) SPADE trees are shown for the 5 healthy donors and 5 septic shock patients. Node size represents cell number and node color is associated with the proportion among all cells from the sample. Thus, cell abundance differences between samples can be visually noted.

**
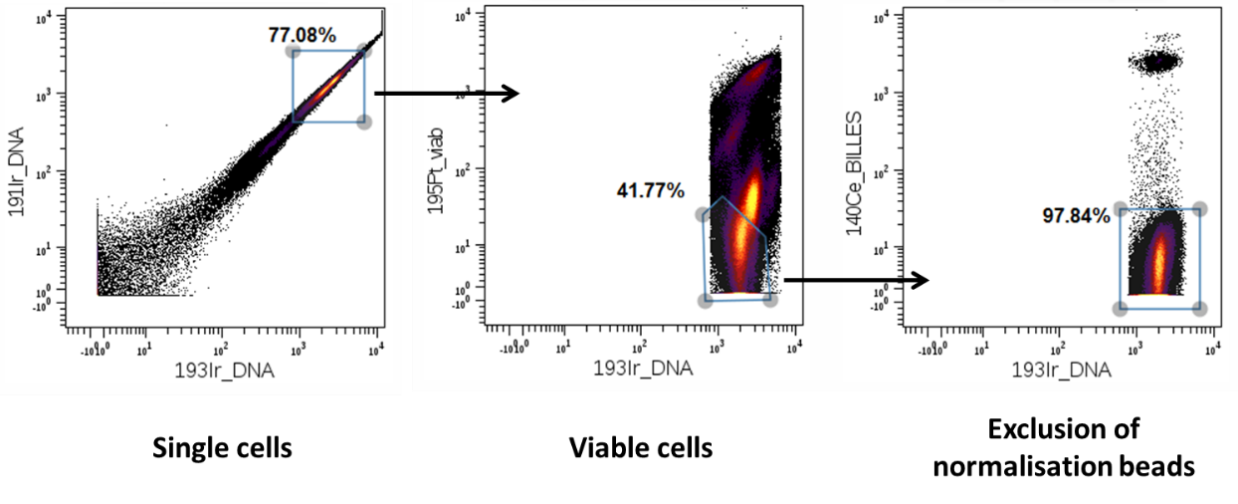
a**

**
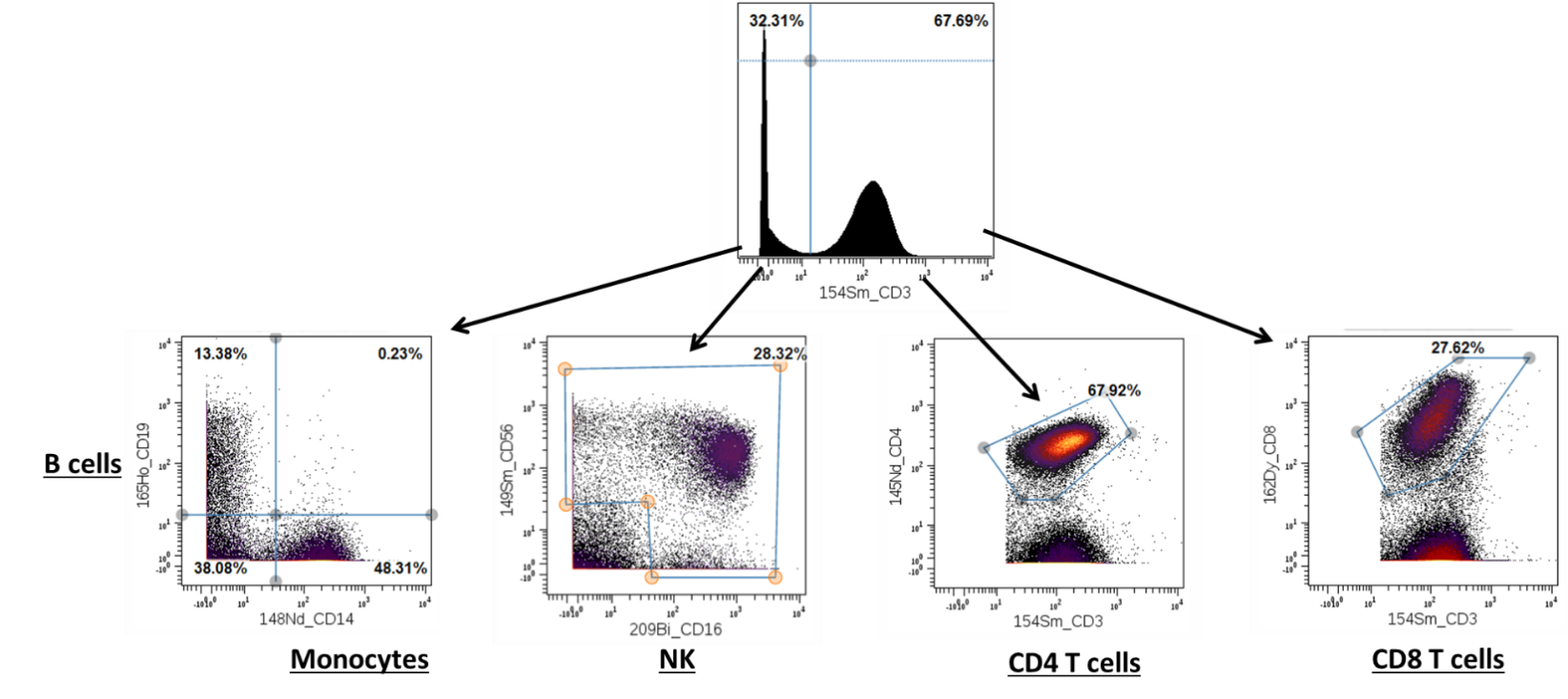
b**

**Supplementary Figure S2. Gating strategy for CyTOF analysis**

(**A**) After CyTOF analysis, events were selected as followed: singulets were identified as ^191^Ir^+^ ^193^Ir^+^ events; then viable cells as ^195^Pt negative events; finally normalization beads were excluded by gating on 140Ce negative events. (**B**) For PBMC subpopulations analysis, cells were first gated according to their expression of CD3. CD4 and CD8 T cells were identified within CD3^+^ cells, while CD19^+^, CD14^+^ and CD16^+^/CD56^+^ non-T cells were used to classify B cells, monocytes and NK cells respectively.
